# *Rickettsia raoultii* in *Haemaphysalis erinacei* from marbled polecats, China–Kazakhstan border

**DOI:** 10.1186/s13071-015-1065-1

**Published:** 2015-09-17

**Authors:** Li-Ping Guo, Lu-Meng Mu, Jun Xu, Su-Hua Jiang, An-Dong Wang, Chuang-Fu Chen, Gang Guo, Wan-Jiang Zhang, Yuan-Zhi Wang

**Affiliations:** School of Medicine, Shihezi University, Shihezi, 832000 China; Xinjiang Entry-Exit Inspection and Quarantine Bureau, Urumqi, 830063 China; College of Animal Science and Technology, Shihezi University, Shihezi, 832000 China

**Keywords:** *Rickettsia raoultii*, PCR, *Haemaphysalis erinacei*, Marbled polecats, China-Kazakhstan border

## Abstract

We found *Rickettsia raoultii* DNA in 2 out of 32 (6.25 %) *Haemaphysalis erinacei* ticks. Result showed that the sequences of five genes (*17*-*kDa*, *gltA*, *ompA*, *rrs*, and *ompB*) were 100 % identity with that of *R. Raoultii* in GenBank. This study is the first report on the presence of *R. raoultii* in *H. erinacei* from wild marbled polecat, *Vormela peregusna*. Our findings suggest that *H. erinacei* parasitizing wild marbled polecat may serve as reservoir and carriers for *R. raoultii* in areas around the China-Kazakhstan border. The transmission of tick-borne diseases originated from wildlife should not be underestimated in border region.

## Findings

In China, at least five validated spot fever group (SFG) rickettsial species have been detected in ticks, including *Rickettsia heilongjiangii*, *R. sibirica* [1], *R. raoultii*, *R. slovaca* [2] and *R. felis* [3]. Of these five rickettsial species, none has been identified in the tick *Haemaphysalis erinacei*. Although no published evidence indicates that *H. erinacei* ticks bites humans, this species is interesting because it coexists with various animal species, including the hedgehog *Hemiechinus auritus* and the marbled polecat, *Vormela peregusna* [4], the later is listed as vulnerable globally by the International Union for Conservation of Nature (IUCN) [5]. The marble polecat is distributed from southeast Europe, through southwest and Central Asia, to Mongolia and northern China [6]. In the present study, we determined the presence of *R. raoultii* in *H. erinacei* from marbled polecats in wetlands around Ebinur Lake, northwest China.

Thirty-two adult ticks, 21 (14 male and seven female) from two marbled polecats and 11 (seven male and four female) from three hedgehogs, were collected in wetlands around Ebinur Lake (189 m above sea level; 82°48′51E 45°04′22N) in northwest China in 2014. The ticks were identified morphologically as *H. Erinacei* and the molecular identification of those ticks by using 16S mitochondrial gene results showed that they have a similarity of 90.95 % with that of *H. concinna* (there are no corresponding 16S mitochondrial gene sequence for *H. Erinacei* in GenBank)*.* The sequences obtained were deposited in GenBank [GenBank: KR053302-KR053305]. Genomic DNA was extracted from individual specimens by using a TIANamp Genomic DNA Kit (TIANGEN, Beijing, China). A targeting gene fragment (434 bp) from the *Rickettsia*-specific 17-kDa surface antigen gene was amplified by PCR following a previously published methodology [7]. Another five genetic markers [1332-, 1060-, 488-, 491-, and 812-bp products of the genes encoding 16S rRNA (*rrs*), citrate synthase (*gltA*), surface cell antigen 1 (*sca1*), and outer membrane proteins A and B (*ompA* and *ompB*)] were amplified by using primers previously described to detect *Rickettsia* spp. in *H. erinacei* [8]. The PCR products were sequenced and phylogenetically analyzed to certify the taxonomic identification of the rickettsial agent.

**Fig. 1:**
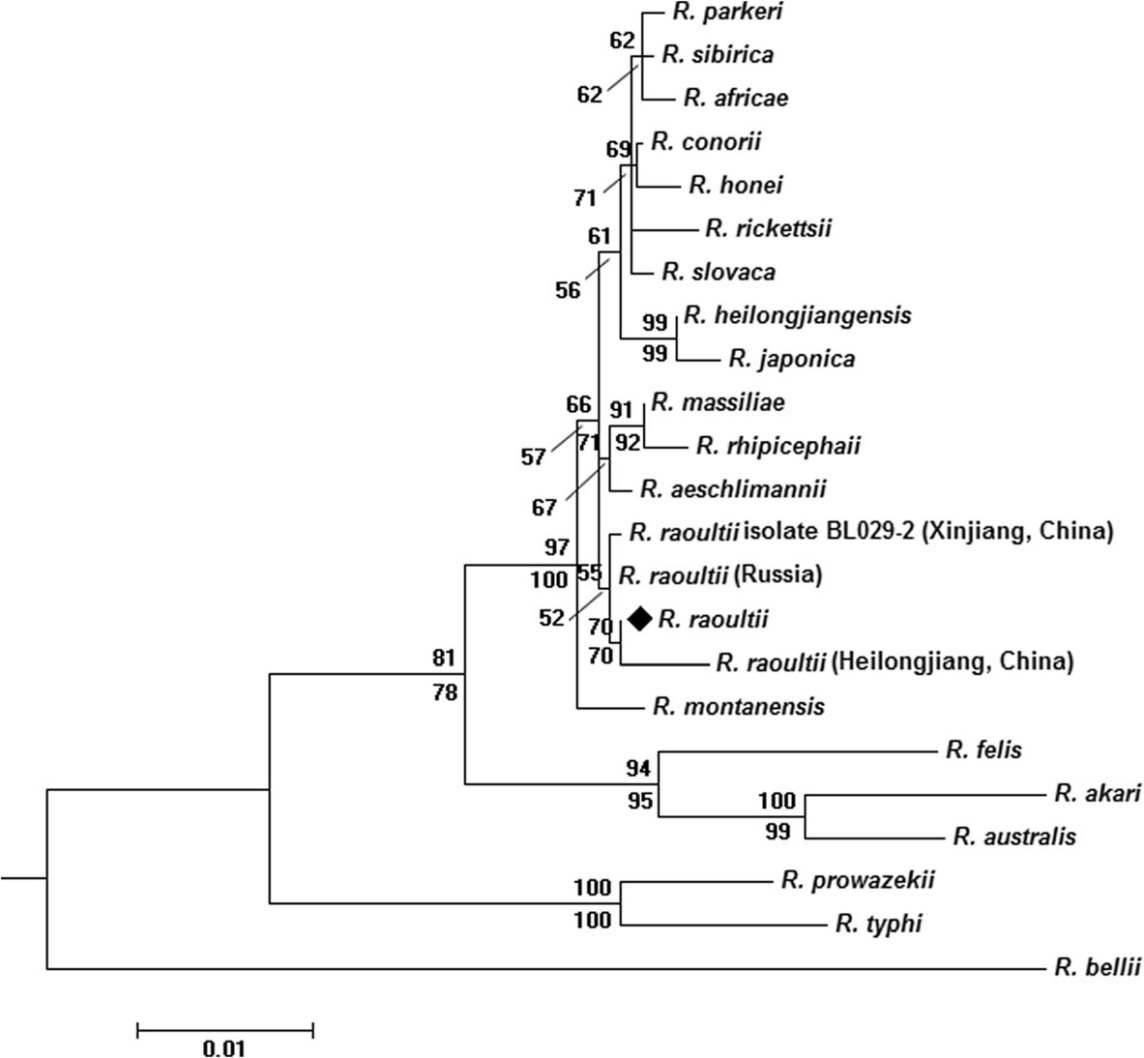
Phylogenetic tree of *17-kDa-ompA-gltA-rrs-sca1-ompB* concatenated sequence of *Rickettsia raoultii * in *Haemaphysalis erinacei* (◆). The tree was constructed on the basis of Maximum Likelihood (Bootstrap replicates: 1000) and Neighbor-Joining (Bootstrap replicates: 500) analyses of concatenated sequence data of six genes (*17-kDa*-*ompA*-*gltA*-*rrs*-*sca1*-*ompB*) using MEGA6. The sequences of *R. bellii* were used as the outgroup in the concatenated sequence data. The scale bar represents the inferred substitutions per nucleotide site. The relative support for clades in the tree produced from the ML and NJ analyses are indicated above and below branches, respectively

Rickettsial DNA was detected in two (both female) out of 32 (6.25 %) *H. erinacei* ticks, which were collected from the same marbled polecat. No rickettsial agent was found in hedgehogs. The sequences BLAST results showed that these two rickettsial sequences of five genes (*17-kDa*, *gltA*, *ompA*, *rrs*, and *ompB*) were the same, and 100 % identity with that of *R. raoultii*. The *sca1* sequences obtained were closest to that of *R. montanensis* str. OSU 85–930 and *R. montanensis* str. M/5-6, with a sequence similarity of 99.18 % (612 out of 617 bp) (There are no corresponding sequence for *R. raoultii* in GenBank). All of the obtained sequences were deposited in GenBank [GenBank: KR608783-KR608788]. The phylogenetic tree produced from the Maximum Likelihood and Neighbor-Joining analyses of the sequence data for the six genes (*17-kDa*-*ompA*-*gltA*-*rrs*-*sca1*-*ompB*) revealed that the *R. raoultii* obtained from *H. erinacei* was culstered into a clade including “*R. Raoultii* (Heilongjiang, China)”, “*R. raoultii* (Russia)”, and “*R. raoultii* isolate BL029-2 (Xinjiang, China)” (Fig. 1).

Based on the information in GenBank, *R. raoultii* have been detected at least in 13 tick species, namely: *Dermacentor nuttallii*, *D. marginatus*, *D. reticulatus*, *D. silvarum*, *Rhipicephalus pumilio*, *Rh. turanicus*, *H. concinna*, *H. japonica*, *Ixodes persulcatus*, *I. ricinus*, *Amblyomma helvolum*, *Hyalomma asiaticum*, and *Hy. lusitanicum* [9]. However, this study is the first to report the presence of *R. raoultii* in *H. erinacei*. In previous studies, *H. erinacei* has been found in birds, the desert hedgehog *Paraechinus aethiopicus,* the North African hedgehog *Atelerix algirus*, stray dogs, the beech marten *Martes foina*, and the least weasel *Mustela nivalis* [10–13]. Here our sampling site, the Ebinur Lake, is widely known to be a station for thousands of wildlife around the China–Kazakhstan border. Approximately 1 million migratory birds arrive here, which is known to be home every year, and more than 160 wild vertebrate species and 230 bird species inhabit and/or migrate at this region [14]. Another several previous studies gave the strong evidence that *R. raoultii* is common and widespread across wildlife such as wild snakes, rats and Mongolian gazelle [15–17]. Our findings suggest that *H. erinacei* parasitizing wild marbled polecat may serve as reservoirs and carriers for *R. raoultii* in areas around the China-Kazakhstan border. In the future, the transmission of tick-borne diseases originated from wildlife should not be underestimated in border region*.* There is a need for international cooperation to survey this and other tick-borne pathogens in migratory birds and wildlife.
